# Down-regulation of zinc finger protein 335 undermines natural killer cell function in mouse colitis-associated colorectal carcinoma

**DOI:** 10.1016/j.heliyon.2024.e25721

**Published:** 2024-02-07

**Authors:** Bin Jiang, Hongjian Zhou, Xingwang Xie, Tian Xia, Chao Ke

**Affiliations:** The Department of Gastrointestinal, Hernia, and Abdominal Wall Surgery, Wuhan Third Hospital (Tongren Hospital of Wuhan University), Wuhan, Hubei Province, 430060, China

**Keywords:** Zinc finger protein 335, Natural killer cells, Colorectal carcinoma, Tumor immunity, Cytotoxicity

## Abstract

Natural killer (NK) cells constitute an active and potent anti-tumor effector population against multiple malignancies. NK cells exploit tumoricidal machinery to restrain colorectal carcinoma (CRC) expansion and invasion. Nonetheless, it is becoming increasingly evident that functional exhaustion considerably compromises the potency of NK cells in patients with CRC. To elucidate the factors that impair NK cell function in the context of CRC, we determined the role of zinc finger protein 335 (ZFP335) in modulating NK cell activity in mouse CRC induced by azoxymethane and dextran sulfate sodium. ZFP335 was profoundly decreased in NK cells in mesenteric lymph nodes of CRC-bearing mice. ZFP335 was especially diminished in NK cells that were both phenotypically and functionally exhausted. Besides, effective ZFP335 knockdown markedly undermined NK cell proliferation, tumoricidal protein production, degranulation, and cytotoxic efficacy on malignant cells, strongly suggesting that ZFP335 reinforces NK cell function. Importantly, ZFP335 knockdown lowered the expression of Janus kinase 1 (JAK1) and Janus kinase 3 (JAK3), both of which play crucial roles in NK cell homeostasis and activation. Collectively, ZFP335 down-regulation is essential for NK cell exhaustion in mesenteric lymph nodes of mice with CRC. We discovered a new ZFP335-JAK1/3 signaling pathway that modulates NK cell exhaustion.

## 1 Introduction

Most colorectal carcinoma (CRC) cases feature uncontrolled growth of epithelial cells of the colorectal mucosa [1]. During CRC development, tumor immunity is mounted to restrain CRC expansion and invasion [2]. Natural killer (NK) cells efficiently kill tumor cells by secreting cytotoxic granules and expressing death ligands [3,4]. Notably, NK cells directly destroy CRC cells and NK cell infiltrates in CRC masses are positively associated with improved clinical outcomes in patients with CRC [5,6]. It is therefore not surprising that *in vivo* modification of NK cell activity and adoptive transfer of genetically engineered NK cells become the most emerging immunotherapy technologies for CRC [7,8]. Nonetheless, the immunosuppressive tumor microenvironment would cause substantial NK cell dysfunction by a variety of mechanisms including triggering NK cell exhaustion [9]. Exhausted NK cells feature slower proliferation, limited production of cytotoxic granules and cytokines, lower expression of activating receptors along with abundant inhibitory receptors, and compromised tumor-killing efficiency [10]. In patients with CRC, degranulation and cytokine expression of tumor-associated NK cells are profoundly impaired, along with down-regulated activating receptors [11], suggesting CRC-induced NK cell exhaustion. Although previous research has implicated that hypoxia, mesenchymal stem cells, tumor cells, regulatory T cells, and myeloid-derived suppressor cells could induce NK cell exhaustion through multiple signaling pathways in the tumor microenvironment [12], the mechanisms responsible for NK cell dysfunction in the CRC microenvironment remain largely elusive.

Zinc finger protein 335 (ZFP335) is a member of the C2H2 zinc finger family [13]. Some C2H2 zinc finger proteins act as transcriptional modulators to control immune responses including NK cell reactivity [14]. Additionally, they may interact with RNA or proteins to regulate target gene expression [13,15]. Early studies reveal that ZFP335 forms complexes with NRC and some histone methyltransferase factors to regulate gene expression [16,17]. ZFP335 can directly bind to DNA and promote transcription via recognizing two consensus DNA motifs [18]. Recent research implies that ZFP335 is necessary for transcriptional programming of T cell precursors and the formation of memory CD8^+^ T cells [ [[19], [20], [21]]]. However, the importance of ZFP335 for NK cell function remains unidentified.

In this research, we noticed that ZFP335 expression was remarkably decreased in NK cells with the exhaustion phenotype in mesenteric lymph nodes (mLN) of mice with chemical-induced CRC. Further experiments revealed that ZFP335 knockdown impaired NK cell proliferation, cytotoxic protein production, and degranulation, strongly suggesting that ZFP335 positively regulates NK cell function probably through the Janus kinase signaling.

## 2 Materials and methods

### 2.1 Animal model

The Wuhan Third Hospital Animal Care and Use Committee approved the experiment procedures that were in accordance with the Animal Research: Reporting of In Vivo Experiments (ARRIVE) guidelines (Approval ID: WTH20220114). 2.5 mg/ml azoxymethane (AOM, Sigma-Aldrich, A5486) was prepared in saline. 220–260 μg of AOM (10 mg/kg) was administered into the peritoneal cavity of each male C57BL/6 J mouse (eight-week-old, 22–26 g) after anesthesia with 3% isoflurane. One week later, each animal was given drinking water containing 2% dextran sulfate sodium (DSS, MW = 36,000–50,000, Sigma-Aldrich, D8906-50G) for one week and then sterile water for the following 2 weeks. This DSS-normal water treatment cycle was performed 3 times in total. Immediately after the last cycle of DSS treatment, the mice were fed with sterile water for 2 weeks (3 months after AOM administration) or 6 weeks (4 months after AOM administration) before they were sacrificed by CO_2_ inhalation. The presence of CRC masses was inspected on each mouse and only those with CRC formation were subjected to further analysis (Supplementary Fig. 1). Mice that were not treated with AOM and DSS were set as healthy controls. To determine the effect of DSS treatment alone, some mice received the same 3 cycles of DSS treatment without the initial AOM administration.

### 2.2 Collecting cells from mLNs

Gut-draining mLNs were resected and placed in a 70-μm cell strainer within a Petri dish containing 1 ml of ice-cold phosphate-buffered saline (PBS). The mLNs were carefully ground using a 3CC syringe plunger until the tissues were dissociated to be single cells. The single-cell suspension was placed in a 15-ml tube and centrifuged at 300× *g* for 3 min. The supernatant was aspirated and the cell pellet was re-suspended in 1 ml of ACK lysis buffer (Thermo Fisher) for 3 min to remove red blood cells, followed by adding 4 ml of PBS to stop lysis. Cells were centrifuged at 300×*g* for 5 min, followed by aspirating the supernatant and re-suspending cells in PBS.

### 2.3 Flow cytometry

A BD FACSCalibur™ cytometer was used for cell phenotype analysis and a BD Influx cell sorter was used for enriching cells of interest. Cell apoptosis was measured using the Apoptosis Detection Kit (Biolegend, 640930) abiding by the supplier's brochure. To discern different cell populations, cell density was adjusted to 0.5–1 × 10^6^/ml in PBS, and corresponding fluorophore-associated antibodies (2 μg/ml each, Supplementary Table 1) were added to incubate cells at 4 °C for 15 min. When quantifying perforin, granzyme B, and signaling molecules (including their phosphorylated forms), cells were centrifuged at 300×*g* for 5 min and the cell pellet was re-suspended in 200 μl of 4% paraformaldehyde-PBS for 20 min at room temperature. After that, cells were centrifuged at 500×*g* for 5 min and the cell pellet was re-suspended in 200 μl of 90% methanol-PBS for 30 min on ice for permeabilization. After centrifugation at 500×*g* for 5 min, the cell pellet was re-suspended in PBS containing the relevant antibodies (2 μg/ml each) for 60 min. To appraise cytokine levels in lentivirus-transduced NK cells, 2 μM monensin (Sigma-Aldrich) plus 2.5 mg/ml brefeldin A (Sigma-Aldrich) was used to incubate NK cells for 4 h immediately after lentiviral transduction to prevent cytokine secretion, followed by above-mentioned procedures of fixation, permeabilization, and antibody incubation.

### 2.4 Lentivirus transduction into NK cells

The scramble control plasmid and the lentiviral plasmid containing a ZFP335 shRNA sequence plus a green fluorescence protein (GFP) coding sequence (TL519897V) were provided by OriGene Wuxi Biotechnology Co., Ltd. Lentiviruses were produced, purified, and titrated by AtaGenix. The MagniSort™ Mouse NK cell Enrichment Kit (Invitrogen) was used to harvest splenic NK cells from healthy C57BL/6 J mice. Before transduction, 5 × 10^5^ NK cells in 500 μl of RPMI 1640 containing 10% fetal bovine serum (FBS) and 250 U/ml IL-2 (R&D Systems) were seeded in a well of a 24-well culture plate (Corning). After 24-h culture, lentiviruses (multiplicity of transduction = 50) and 6 μg/ml polybrene (Sigma-Aldrich) were added to the culture, followed by 1-h centrifugation at 700×*g* at 32 °C. The cells were then cultured in an incubator for 16 h, followed by incubation in the same volume of fresh media containing 250 U/ml IL-2 for another 72 h. GFP^+^ cells were regarded as successfully transduced NK cells.

### 2.5 Tumoricidal capacity assay

1 × 10^5^ NK cells and 2 × 10^4^ YAC-1 lymphoma cells (Procell Biotech) were mixed and incubated at 37 °C for 4 h. The cell mixture was transferred into an Eppendorf tube for 5-min centrifugation at 200×*g*. To distinguish NK cells in the mixture, the whole cells were incubated for 15 min in 100 μl of ice-cold PBS containing allophycocyanin-cyanine-7-conjugated NK1.1 antibody, followed by 15-min incubation in 100 μl of ice-cold PBS containing phycoerythrin-cyanine-7-conjugated CD107a antibody for degranulation detection or apoptosis assessment using the Apoptosis Detection Kit as described previously.

### 2.6 Reverse transcription (RT) and quantitative polymerase chain reaction (qPCR)

Cells were subjected to RNA extraction following the manual of the PicoPure RNA Isolation Kit (Applied Biosystems). mRNAs were reversely transcribed to cDNAs using the oligo (dT), dNTPs, and the reverse transcriptase provided in the FastKing RT Kit (Tiangen Biotech). cDNAs were subjected to qPCR using 2 × SYBR Green, AmpliTaq Gold™ DNA polymerase, dNTPs, and the passive reference dye provided in the SYBR® Green Universal Master Mix (Applied Biosystems). The reaction was executed in a StepOnePlus™ Real-Time PCR Thermocycler (Applied Biosystems). Primers for amplifying target genes are shown in Supplementary Table 2.

### 2.7 Western blot

Cells were centrifuged at 500×*g* for 5 min and the supernatant was discarded. The cell pellet was re-suspended in cold RIPA extraction buffer (Beyotime Inc, 100 μl for 1 × 10^6^ cells) in the presence of protease inhibitor cocktail and phosphatase inhibitor cocktail (Beyotime Inc). After staying on ice for 30 min, the lysate was sonicated for 30 s with 50% pulse. The lysate was centrifuged at 12,000×*g* for 15 min and the protein-containing supernatant was collected into a new tube. Extracted proteins were quantified using the Micro BCA™ Protein Assay Kit (Thermo Fisher). Each sample containing 10 μg protein was subjected to electrophoresis, transfer, antibody incubation, and chemiluminescent detection. The polyclonal ZFP335 antibody (TA345356, 1:1000) was obtained from OriGene Wuxi Biotechnology Co., Ltd. The JAK1 antibody (MA5-32780, 1:1000), AK3 antibody (MA5-15561, 1:1000), and β-Actin antibody (MA1-140, 1:2000) were purchased from Invitrogen. A Biospetrum 300 (UVP Ltd) was applied for imaging and signal quantification.

### 2.8 Statistics

GraphPad Prism 9.0.0. was utilized for data graphing and statistical evaluation. The results are indicated as mean ± SD. Student's *t*-test was used for two-group comparisons while Welch ANOVA was used for multiple comparisons. A *P* value < 0.05 is regarded as statistically significant.

## 3 Results

### 3.1 ZFP335 expression in mLN NK cells in mice with CRC

**Fig. 1:**
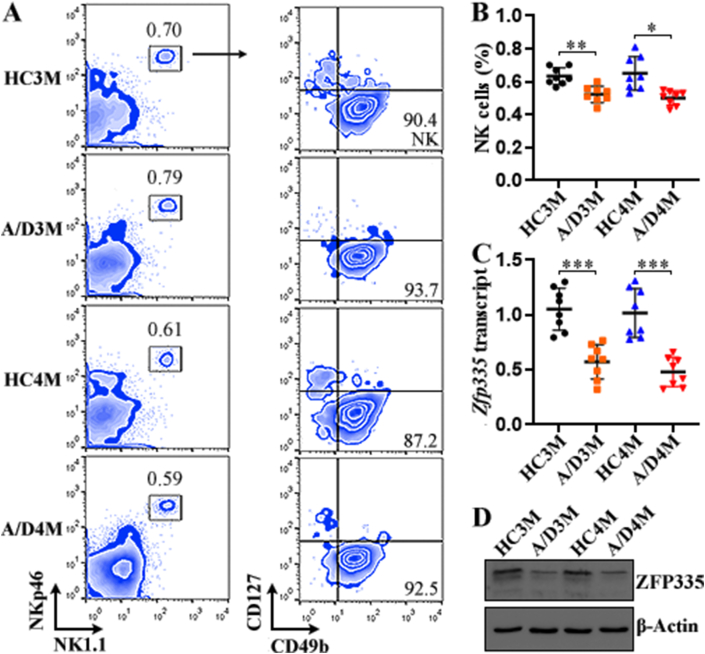
ZFP335 expression in mLN NK cells. (A) Zebra plots indicate NKp46^+^NK1.1^+^CD127^−^CD49b^+^ NK cells in mLNs. HC3M: healthy mice at month 3. A/D3M: 3 months after AOM/DSS treatment. HC4M: healthy mice at month 4. A/D4M: 4 months after AOM/DSS treatment. **(B)** Percentage of mLN NK cells in total lymphocytes. **(C)***Zfp335* transcript in mLN NK cells. N = 8 mice per group in three experiments. **(D)** ZFP335 protein in mLN NK cells. Each sample contains NK cell proteins pooled from 20 mice. *: *P* < 0.05. ***: *P* < 0.001. Welch ANOVA.

The AOM-and-DSS treatment triggered observable CRC development in mice (Supplementary Fig. 1). mLN cells were resected from the control mice or mice with CRC and processed to recognize NK cells on a flow cytometer. After excluding cell clusters and gating viable lymphocytes (Supplementary Fig. 2), a population co-expressing NKp46 and NK1.1 was discriminated among lymphocytes (Fig. 1A). Within this population, four subpopulations were observed according to the expression of two surface markers, i.e. CD49b and CD127 (Fig. 1A). Because CD127 is primarily expressed on immature B cells, mature T cells, and innate lymphoid cells (ILCs) whereas CD49b is highly expressed by NK cells [ [[22], [23], [24], [25]]], we considered CD127^−^CD49b^+^ cells as bona fide NK cells (Fig. 1A). The proportion of NK cells was significantly decreased in mice with CRC at each time point, in comparison with healthy mice (Fig. 1B). To examine whether mLN NK cell maturation status was altered after CRC induction, we tested the expression of NK cell development markers such as CD27, CD11b, CD43, and Ly49A. As exhibited in Supplementary Figs. 3A–3D, the expression profiles of CD27 and CD11b were comparable between healthy mice and CRC-bearing mice. Similarly, the frequencies of CD43^-^Ly49A^−^, CD43^-^Ly49A^+^, and CD43^+^Ly49A^+^ NK cells were also comparable between healthy mice and CRC-bearing mice. (Supplementary Figs. 3E–3H). Therefore, the majority of mLN NK cells were mature NK cells (probably stage E and F) in both healthy mice and CRC-bearing mice. Interestingly, in mice with CRC, mLN NK cells down-regulated *Zfp3*35 mRNA by around 50% in comparison to their counterparts in healthy mice (Fig. 1C). ZFP335 down-regulation was substantiated by Western blot (Fig. 1D and Supplementary Fig. 1).

### 3.2 ZFP335 expression in mLN NK cells with the exhaustion phenotype

**Fig. 2:**
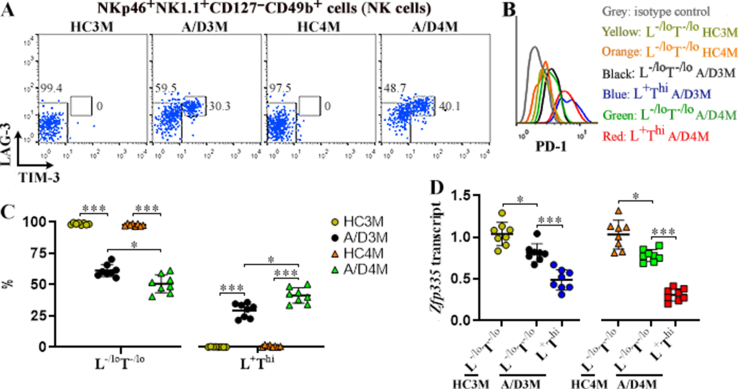
ZFP335 expression in NK subsets. (A) Dot plots indicate TIM-3 and LAG-3 expression on the surface of NKp46^+^NK1.1^+^CD127^−^CD49b^+^ mLN NK cells. HC3M: healthy mice at month 3. A/D3M: 3 months after AOM/DSS treatment. HC4M: healthy mice at month 4. A/D4M: 4 months after AOM/DSS treatment. **(B)** PD-1 staining on each NK subset. L^−/lo^T^−/lo^: LAG-3^-/lo^TIM-3^-/lo^ NK cells. L ^+^ T^hi^: LAG-3^+^TIM-3^hi^ NK cells. The image represents two independent experiments. **(C)** Proportions of LAG-3^-/lo^TIM-3^-/lo^ and LAG-3^+^TIM-3^hi^ NK cells. **(D)***Zfp335* transcript in the two NK subsets. Note that LAG-3^+^TIM-3^hi^ NK cells in healthy mouse mLNs are absent. N = 8 mice per group in three experiments. *: *P* < 0.05. ***: *P* < 0.001. Welch ANOVA.

Lymphocyte-activation protein-3 (LAG-3) and T-cell immunoglobulin and mucin domain-3 (TIM-3) are well-accepted exhaustion markers [10]. We appraised their expression on the surface of NKp46^+^NK1.1^+^CD127^−^CD49b^+^ cells, i.e. mLN NK cells. Normal mLN NK cells did not express LAG-3 or TIM-3, whereas mLN NK cells of tumor-bearing mice expressed higher LAG-3 and TIM-3 to generate two NK subsets: LAG-3^+^TIM-3^hi^ NK cells and LAG-3^-/lo^TIM-3^-/lo^ NK cells (Fig. 2A). The former also expressed higher programmed death-1 (another NK cell exhaustion marker) than the latter (Fig. 2B). Notably, the proportion of LAG-3^+^TIM-3^hi^ NK cells was increased from the 3rd month to 4th month after the initial AOM administration (Fig. 2C), suggesting an increase in exhausted NK cells during CRC development. Interestingly, both LAG-3^+^TIM-3^hi^ NK cells and LAG-3^-/lo^TIM-3^-/lo^ NK cells down-regulated *Zfp335* transcript in CRC-bearing mice, in comparison to LAG-3^-/lo^TIM-3^-/lo^ NK cells in normal mice (Fig. 2D). Of note, LAG-3^+^TIM-3^hi^ NK cells expressed the lowest level of *Zfp335* transcript (Fig. 2D).

**Fig. 3:**
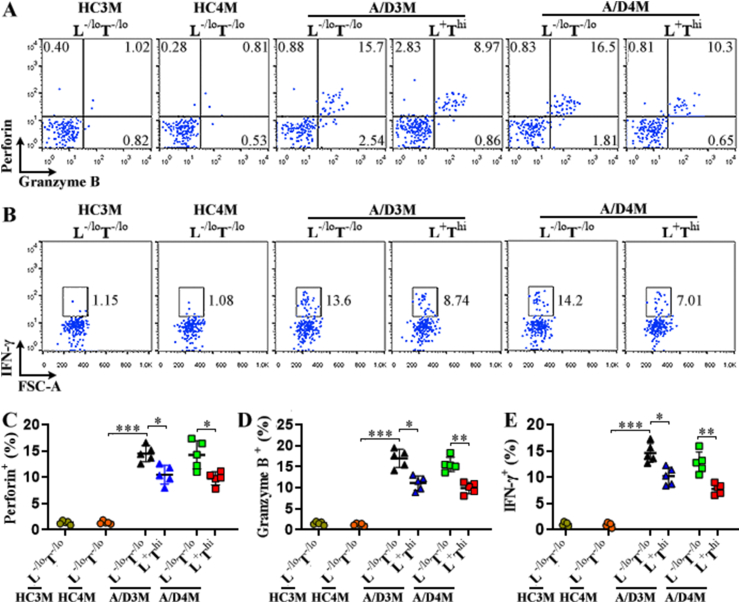
Cytotoxic proteins in mLN NK subsets. (A) Dot plots show intracellular perforin and granzyme B in mLN NK cell subsets. L^−/lo^T^−/lo^: LAG-3^-/lo^TIM-3^-/lo^ NK cells. L ^+^ T^hi^: LAG-3^+^TIM-3^hi^ NK cells. HC3M: healthy mice at month 3. A/D3M: 3 months after AOM/DSS treatment. HC4M: healthy mice at month 4. A/D4M: 4 months after AOM/DSS treatment. **(B)** Dot plots show intracellular IFN-γ. **(C to E)** Percentages of perforin^+^ (C), granzyme B^+^ (D), and IFN-γ^+^ (E) cells in each NK subset. N = 5 mice per group in three experiments. *: *P* < 0.05. **: *P* < 0.01. ***: *P* < 0.001. NS: not significant. Welch ANOVA.

Next, we analyzed the expression of cytotoxic perforin and granzyme B to reveal the functions of the two NK subsets. As shown in Fig. 3A, C, and 3D, perforin and granzyme B were scarcely expressed in mLN NK cells of normal mice. In mice with CRC, either LAG-3^+^TIM-3^hi^ NK cells or LAG-3^-/lo^TIM-3^-/lo^ NK cells significantly produced these cytotoxic mediators, suggesting their activation status. Notably, the LAG-3^-/lo^TIM-3^-/lo^ subset produced remarkably higher perforin and granzyme B than the LAG-3^+^TIM-3^hi^ subset. Furthermore, intracellular IFN-γ expression showed similar changes in the two NK subsets (Fig. 3B and E). Therefore, LAG-3^+^TIM-3^hi^ NK cells were likely exhausted NK cells compared with LAG-3^-/lo^TIM-3^-/lo^ NK cells.

### 3.3 DSS treatment alone cannot induce ZFP335 down-regulation and NK cell exhaustion

To determine whether DSS treatment alone can cause the above-mentioned changes, some mice were fed with 2% DSS in the same way as described above but without AOM injection (Supplementary Fig. 4A). mLN NK cells in these mice were then analyzed. As shown in Supplementary Figs. 4B and 4C, DSS treatment did not significantly alter NK cell frequency. Besides, DSS treatment had no effect on *Zfp335* transcript expression in mLN NK cells (Supplementary Fig. 4D). NK cells still expressed little LAG-3 and TIM-3 after DSS treatment (Supplementary Fig. 4E). DSS treatment resulted in a decrease in colon length, suggesting the onset of chronic colitis (Supplementary Fig. 4F). DSS treatment did not influence the production of perforin and granzyme B compared with the control group (Supplementary Fig. 5). Therefore, ZFP335 down-regulation and NK cell exhaustion might be highly associated with CRC rather than chronic colitis.

### 3.4 ZFP335 silencing undermines NK cell function

**Fig. 4:**
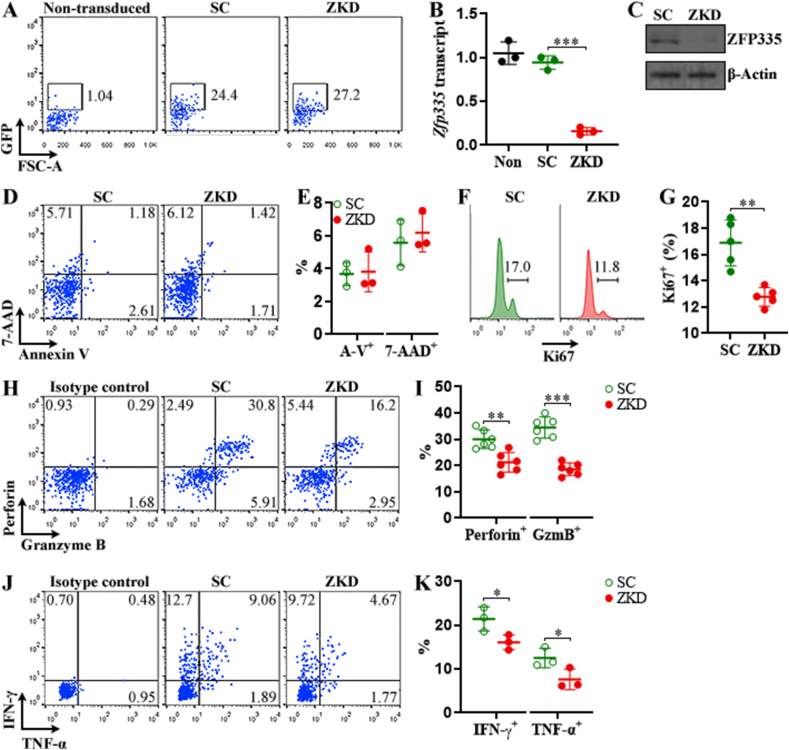
ZFP335 silencing-induced changes in NK cell function. (A) Dot plots show GFP expression in splenic NK cells on day 3 after lentiviral transduction. Non-transduced: no lentivirus. SC: transduction with scramble shRNA lentivirus. ZKD: transduction with ZFP335 shRNA lentivirus. **(B)***Zfp335* transcript in GFP ^+^ NK cells on post-transduction day 3. **(C)** ZFP335 protein in GFP^+^ NK cells on post-transduction day 3. **(D and E)** Apoptosis and necrosis of GFP^+^ NK cells. Representative plots are shown in (D) and statistics are shown in (E). **(F and G)** Ki67 staining in GFP^+^ NK cells on post-transduction day 3. Representative histograms are shown in (F) and statistics of Ki67^+^ cells are shown in (G). **(H)** Dot plots indicate intracellular perforin and granzyme B in GFP^+^ NK cells. **(I)** Percentages of perforin^+^ and granzyme B^+^ cells. **(J)** Dot plots indicate intracellular IFN-γ and TNF-α in GFP^+^ NK cells. In (H & J), cells were treated with 2.5 mg/ml brefeldin A and 2 μM monensin for 4 h before intracellular staining. **(K)** Percentages of IFN-γ^+^ and TNF-α^+^ cells. N = 3 or 6 independent samples per group in three experiments. *: *P* < 0.05. **: *P* < 0.01. ***: *P* < 0.001. Student's *t*-test.

To elucidate ZFP335 function, we harvested splenic NK cells from normal C57BL/6 J mice and infected them with control lentivirus or ZFP335 shRNA lentivirus. We designated control lentivirus-transduced NK cells as SC NK whereas ZFP335 shRNA lentivirus-transduced NK cells were designated as ZKD NK. Because the lentiviruses also encoded GFP, the proportion of GFP^+^ NK cells signified transduction efficiency. On post-transduction day 3, GFP^+^ NK cell frequency reached approximately 25% (Fig. 4A). We collected these GFP^+^ NK cells through flow cytometry and used them in further analysis. ZFP335 silencing was corroborated by profound down-regulation of both *Zfp335* transcript and ZFP335 protein in GFP ^+^ NK cells (Fig. 4B and C, and Supplementary Fig. 6A). ZFP335 silencing did not profoundly influence NK cell apoptosis or necrosis (Fig. 4D and E). ZFP335 silencing did not impact NK cell phenotype or maturation, as evidenced by no change in TIM-3, LAG-3, CD27, CD11b, CD43, and Ly49A on NK cell surface (Supplementary Figs. 6B and 7). Because these NK cells were activated by IL-2 before and after transduction, we quantified GFP^+^ NK cell expansion and cytotoxic mediator expression. As indicated in Fig. 4F and G, fewer Ki67 were observed in ZKD NK compared with SC NK, suggesting slower proliferation. Besides, perforin, granzyme B, TNF-α, and IFN-γ were all down-regulated in ZKD NK compared with SC NK (Fig. 4H–K), strongly implying that ZFP335 is essential for IL-2-mediated activation of NK cells.

**Fig. 5:**
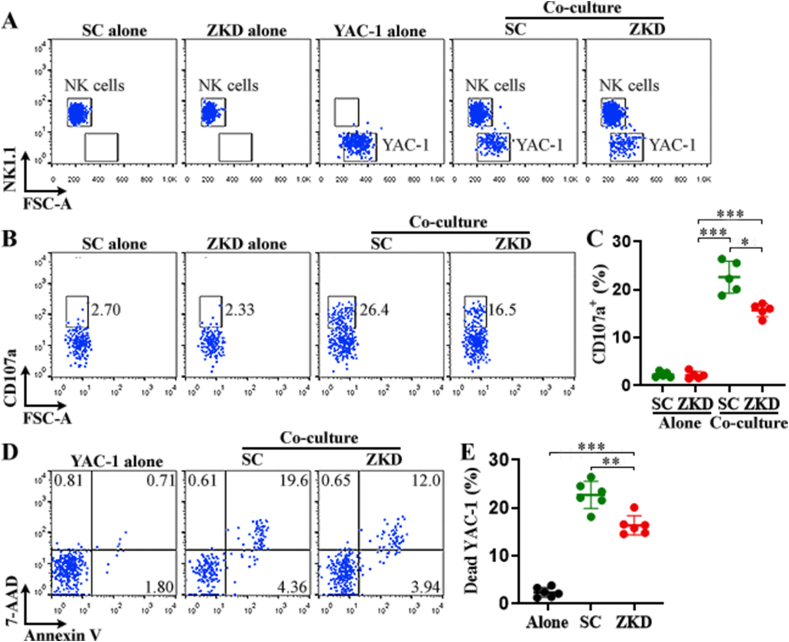
NK cell degranulation and cytotoxicity. (A) Dot plots show NK1.1^+^ NK cells and NK1.1^-^ YAC-1 cells in the culture. Please note that APC/Cy7 anti-NK1.1 antibody was used to stain NK cells because GFP expression in NK cells was not strong enough to precisely separate NK cells and YAC-1 cells. SC alone: SC NK cells alone. ZKD alone: ZKD NK cells alone. YAC-1 alone: YAC-1 cells alone. Co-culture: NK cells with YAC-1 cells. **(B)** Dot plots show CD107a expression on NK cells. **(C)** Percentage of CD107a^+^ NK cells. **(D)** Dot plots show YAC-1 cell apoptosis and necrosis. **(E)** Percentage of dead (necrotic + apoptotic) YAC-1 cells. N = 5 or 6 independent samples per group in two experiments. *: *P* < 0.05. **: *P* < 0.01. ***: *P* < 0.001. Welch ANOVA.

To measure tumoricidal efficacy, we incubated GFP^+^ NK cells along with YAC-1 cells (effector: target = 5:1) for 4 h (Fig. 5A). After that, we detected CD107a, which is a degranulation marker, on NK cell surface. Meanwhile, we evaluated YAC-1 apoptosis. As shown in Fig. 5B and C, CD107a expression was considerably increased on both SC NK and ZKD NK after the co-culture. However, lower CD107a was observed on ZKD NK compared with SC NK, suggesting weaker degranulation of ZKD NK. Consistently, ZKD NK were less cytotoxic because they resulted in fewer dead YAC-1 cells compared with SC NK (Fig. 5D and E).

### 3.5 ZFP335 silencing reduces JAK1/3

**Fig. 6:**
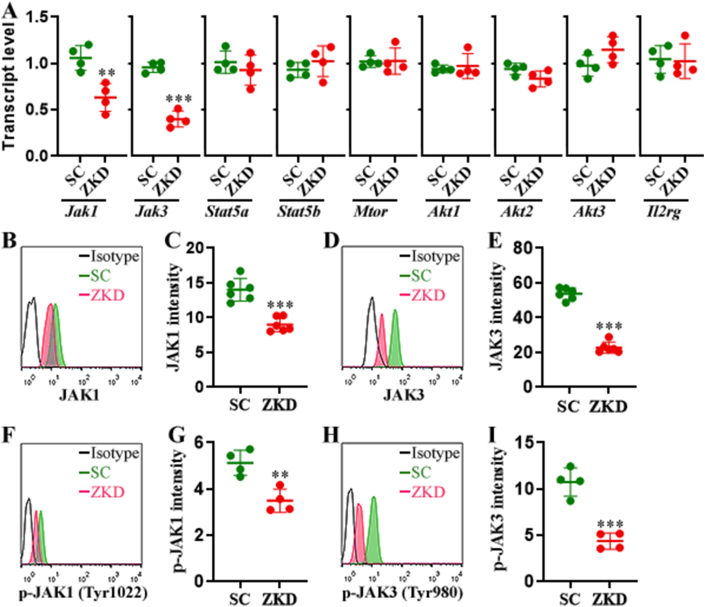
ZFP335 silencing-induced changes in signaling molecule expression. (A) Transcript levels of indicated signaling molecules. SC: SC NK cells. ZKD: ZKD NK cells. **(B and C)** Intracellular JAK1 expression. Representative histograms are shown in (B) and statistics of JAK1 fluorescence intensity are shown in (C). **(D and E)** Intracellular JAK3 expression. Representative histograms are shown in (D) and statistics of JAK3 fluorescence intensity are shown in (E). **(F and G)** Intracellular phosphorylated JAK1. Representative histograms are shown in (F) and statistics of the fluorescence intensity of phosphorylated JAK1 are shown in (G). **(H and I)** Intracellular phosphorylated JAK3. Representative histograms are shown in (H) and statistics of the fluorescence intensity of phosphorylated JAK3 are shown in (I). N = 4 or 6 independent samples per group in two experiments. **: *P* < 0.01. ***: *P* < 0.001. Student's *t*-test.

As a transcription factor, ZFP335 might modulate the expression of IL-2-signaling-associated proteins. To test this hypothesis, we first analyzed the transcripts of JAK1, JAK3, STAT5A, STAT5B, mTOR, AKT1, AKT2, AKT3, and IL-2Rγ, all of which are involved in IL-2-induced NK cell proliferation and cytotoxicity. ZKD NK expressed lower JAK1 and JAK3 than SC NK (Fig. 6A). No significant differences in the transcript levels of other molecules were found. Intracellular staining confirmed down-regulation of JAK1 and JAK3 in ZKD NK cells relative to SC NK cells, with JAK3 exhibiting a more profound decrease (by 60%) than JAK1 (by 35%) (Fig. 6B–E). The changes in JAK1 and JAK3 were corroborated by Western blot (Supplementary Fig. 8). Accordingly, phosphorylated JAK1 and phosphorylated JAK3 were also diminished in ZKD NK cells (Fig. 6F–I), suggesting the down-regulation of IL-2 signaling.

## 4 Discussion

Due to unidentified reasons, it is difficult for NK cells to enter the CRC parenchyma, as evidenced by low frequencies of infiltrating NK cells in CRC tissues [26]. We also noticed limited intratumoral NK cells in mice with CRC in the pilot analysis. We then decided to study mLN NK cells because mLNs are important sites of CRC metastasis [[27], [28], [29]]. We speculated that metastatic CRC cells could form a cancer microenvironment to alter NK cell activity in mLNs. We observed lower ZFP335 expression in exhausted NK cells that co-expressed LAG-3 and TIM-3. As inhibitory receptors, LAG-3 and TIM-3 initiate suppressive signal pathways to weaken NK cell cytotoxicity [30,31]. Indeed, the production of cytotoxic mediators was reduced in LAG-3^+^TIM-3^hi^ NK cells in comparison to LAG-3^-/lo^TIM-3^-/lo^ NK cells, confirming functional exhaustion. Notably, LAG-3^+^TIM-3^hi^ NK cells were progressively increased over time, suggesting that the tumor microenvironment exacerbated NK cell dysfunction. Multiple factors, such as dysregulated signaling, immunosuppressive cells, metabolic dysregulation, and exosomes secreted by tumor cells have been reported to cause NK cell exhaustion [10,32]. Whether these factors are responsible for the observed mLN NK cell exhaustion deserves future analysis.

Another mystery is why ZFP335 is down-regulated in NK cells. To our knowledge, the factors that modulate ZFP335 expression remain largely unknown. Because ZFP335 down-regulation is present in LAG-3^+^TIM-3^hi^ NK cells, ZFP335 expression could be influenced by signal pathways related to one or several inhibitory receptors. In the future, complete revelation of the signal network and transcriptomic alterations within exhausted NK cells could provide valuable clues.

Our study outlined significant decreases in tumoricidal mediators and cytotoxic cytokines in ZFP335-knockdown NK cells, suggesting that ZFP335 supports NK cell function. Nonetheless, whether ZFP335 binds to DNA sequences to impact the expression of these effector factors remains unrevealed. ZFP335 regulates gene transcription by forming complexes with NRC and histone methyltransferase factors or by directly binding DNA [[16], [17], [18]]. The target genes of ZFP335, such as *Bcl6*, *Rorc*, and *Tcf7*, are essential for cell proliferation, cell death, metabolism, mitochondrial function, RNA processing, and transcriptional regulation [ [17,[19], [20], [21]]]. Therefore, ZFP335 down-regulation might elicit multiple biological alterations of NK cells, necessitating future revelation of ZFP335-mediated changes in transcriptomic and proteomic profiles. It is also important to determine the presence of ZFP335-binding motifs in genes key to NK cell function.

Importantly, we found significant down-regulation of JAK1 and JAK3 in ZFP335-knockdown NK cells. JAK1 and JAK3 are essential for the downstream signaling of cytokines key to NK cell development and function, such as IL-2 and IL-7 [33,34]. JAK1 and JAK3 participate in the phosphoinositide 3-kinase signaling and STAT5 signaling to enhance NK cell proliferation, survival, and cytotoxicity [33,34]. Therefore, the down-regulation of JAK1 and JAK3 impairs NK cell function. However, it is unclear whether ZFP335 directly binds to the promoters of *Jak1* and *Jak3*. Our preliminary study did not find ZFP335-binding motifs in the genes of *Jak1* and *Jak3*. Therefore, how ZFP335 regulates JAK1 and JAK3 expression needs to be disclosed in further investigations.

This study still has some limitations. First, the clinical significance of the findings is unclear. The regulatory role of ZFP335 in NK cell activity, especially in mLNs and intestinal mucosa, shall be studied in future clinical research. If ZFP335 has the same effect on human NK cells, it could be targeted for CRC treatment. Second, it is unknown whether ZFP335 down-regulation in NK cells is CRC-specific. NK cell exhaustion is present in various tumors [35]. If ZFP335 expression is modulated by inhibitory signaling pathways associated with TIM-3 and LAG-3, ZFP335 down-regulation in NK cells might occur in other tumors. Third, the relationship between ZFP335 expression and the CRC microenvironment is not determined. In the tumor microenvironment, various scenarios such as prolonged cytokine exposure, frequent contact with malignant cells, or metabolic dysregulation can result in NK cell exhaustion [32]. Whether these factors are involved in ZFP335 down-regulation deserves further research. Fourth, we focused on mLN NK cells because of the rarity of intratumoral NK cells. However, NK cells in other immune tissues/organs such as the spleen, distal lymph nodes, tertiary lymphoid structures, or mucosa-associated lymphoid tissue should be analyzed to understand the systemic profile of NK cell activity. Furthermore, it is unknown whether NK cell exhaustion and ZFP335 down-regulation are ubiquitous in all mLNs. Because the chances of CRC metastasis into different regional lymph nodes are not the same [36,37], it would be important to measure NK cell exhaustion and ZFP335 down-regulation in individual lymph nodes in the future.

In conclusion, ZFP335 reinforces NK cell function probably via the JAK1/3 signaling pathway. ZFP335 down-regulation contributes to the exhaustion of CRC-associated mLN NK cells. Therefore, manipulating ZFP335 expression could be a novel therapeutic strategy for CRC.

## Ethics approval

The Wuhan Third Hospital Animal Care and Use Committee approved the experiment procedures that were in accordance with the Animal Research: Reporting of In Vivo Experiments (ARRIVE) guidelines (Approval ID: WTH20220114).

## Funding

This work was supported by the Wuhan Clinical Research Foundation, China (Grant# WX14C22) and Wuhan Municipal Medical Research Foundation, China (Grant# WX20A07).

## CRediT authorship contribution statement

**Bin Jiang:** Data curation, Investigation, Methodology. **Hongjian Zhou:** Data curation, Investigation. **Xingwang Xie:** Investigation, Methodology, Validation. **Tian Xia:** Data curation, Methodology. **Chao Ke:** Project administration, Supervision, Writing – original draft, Writing – review & editing.

## Declaration of competing interest

The authors declare that they have no known competing financial interests or personal relationships that could have appeared to influence the work reported in this paper.

## Abbreviations

AOM: azoxymethane
CRC: colorectal carcinoma
DSS: dextran sulfate sodium
IFN: interferon
IL: interleukin
JAK: Janus kinase
LAG-3: lymphocyte-activation protein 3
mLN: mesenteric lymph node
NK: natural killer
SIRT6: sirtuin 6
PD-1: programmed death-1
TIM-3: T-cell immunoglobulin and mucin domain 3
TNF: tumor necrosis factor
ZFP335: zinc finger protein 335
