# Supplementary material for: Down-regulation of zinc finger protein 335 undermines natural killer cell function in mouse colitis-associated colorectal carcinoma
Source: Heliyon. 2024 Feb 7;10(4):e25721. doi: 10.1016/j.heliyon.2024.e25721 (PMC10875430; doi:10.1016/j.heliyon.2024.e25721)
Supplement: Multimedia component 1 [file mmc1.docx]

| **Supplementary Table 1. Flow cytometry antibodies** | | |
| --- | --- | --- |
| **Name** | **Clone #** | **Supplier** |
| Brilliant Violet 605 anti-NKp46 | 29A1.4  PK136  A7R34  DX5  C9B7W  B8.2C12  RMP1-30  XMG1.2  MP6-XT22  S16009A  QA16A02  11F6  1D4B  413104  5H2  Jak1Y10221023-F11  JAK3Y980981-E10  Polyclonal | BioLegend |
| APC/Cy7 anti-NK1.1 |  | BioLegend |
| PE/Cy5 anti-CD127 |  | BioLegend |
| FITC anti-CD49b |  | BioLegend |
| PE/Cy7 anti-LAG-3 |  | BioLegend |
| PE anti-TIM-3 |  | BioLegend |
| APC anti-PD-1 |  | BioLegend |
| APC anti-IFN-γ |  | BioLegend |
| PE/Cy5.5 anti-TNF-α |  | BioLegend |
| Pacific blue anti-perforin |  | BioLegend |
| APC anti-granzyme B |  | BioLegend |
| PE anti-Ki67 |  | BioLegend |
| PE/Cy7 anti-CD107a |  | BioLegend |
| Alexa Fluor® 647 anti-JAK1 |  | R&D |
| Anti-JAK3 |  | Invitrogen |
| APC anti-phospho-JAK1 |  | Invitrogen |
| APC anti-phospho-JAK3 |  | Invitrogen |
| APC goat anti-mouse IgG |  | Invitrogen |
| PE anti-CD27 | LG.3A10 | BioLegend |
| APC anti-CD11b | M1/70 | BioLegend |
| PE/Cy7 anti-CD43 | 1B11 | BioLegend |
| PE anti-Ly49A | YE1/48.10.6 | BioLegend |
| Brilliant Violet 605 Rat IgG2a* | RTK2758 | BioLegend |
| APC/Cy7 Mouse IgG2a* | MOPC-173 | BioLegend |
| PE/Cy5 Rat IgG2a* | RTK2758 | BioLegend |
| PE Rat IgG2a* | RTK2758 | BioLegend |
| FITC Rat IgM* | RTK2118 | BioLegend |
| PE/Cy7 Rat IgG1* | G0114F7 | BioLegend |
| PE Rat IgG1* | G0114F7 | BioLegend |
| APC Rat IgG2b* | G013B8 | BioLegend |
| APC Rat IgG1* | G0114F7 | BioLegend |
| PE/Cy5.5 Rat IgG1* | RTK2071 | BioLegend |
| Pacific blue Rat IgG2a* | RTK2758 | BioLegend |
| APC Mouse IgG1* | MOPC-21 | BioLegend |
| PE/Cy7 Rat IgG2a* | RTK2758 | BioLegend |
| Alexa Fluor® 647 Rat IgG2b* | RTK4530 | BioLegend |
| Mouse IgG1* | MOPC-21 | BioLegend |
| APC Rabbit IgG* | EPR25A | Abcam |
| PE Hamster IgG* | HTK888 | BioLegend |
| *** indicates an isotype control** |  |  |

| **Supplementary Table 2. Primers** | | | |
| --- | --- | --- | --- |
| **Gene** | **Forward (5’-3’)** | **Reverse (5’-3’)** | |
| *Zfp335* | GAGGACTTCCTGCTGTCTCATG | | CCACTCCTCAAAGCTGTTGGCA |
| *Jak1* | CTGTCTACTCCATGAGCCAGCT | | CCTCATCCTTGTAGTCCAGCAG |
| *Jak2* | GCTACCAGATGGAAACTGTGCG | | GCCTCTGTAATGTTGGTGAGATC |
| *Stat5a* | CCTGTTTGAGTCTCAGTTCAGCG | | TGGCAGTAGCATTGTGGTCCTG |
| *Stat5b* | CACAGTTCAGCGTCGGTGGAAA | | CTGTGGCATTGTTGTCCTGGCT |
| *Mtor* | AGAAGGGTCTCCAAGGACGACT | | GCAGGACACAAAGGCAGCATTG |
| *Akt1* | GGACTACTTGCACTCCGAGAAG | | CATAGTGGCACCGTCCTTGATC |
| *Akt2* | CCAACACCTTTGTCATACGCTGC | | GCTTCAGACTGTTGGCGACCAT |
| *Akt3* | GAGATGGATGCGTCTACAACCC | | TCCACTTGCCTTCTCTCGAACC |
| *Il2rg* | GGAGCAACAGAGATCGAAGCTG | | CCACAGATTGGGTTATAGCGGC |
| *Actb* | GATGGTGAAGGTCGGTGTGA | | TGAACTTGCCGTGGGTAGAG |


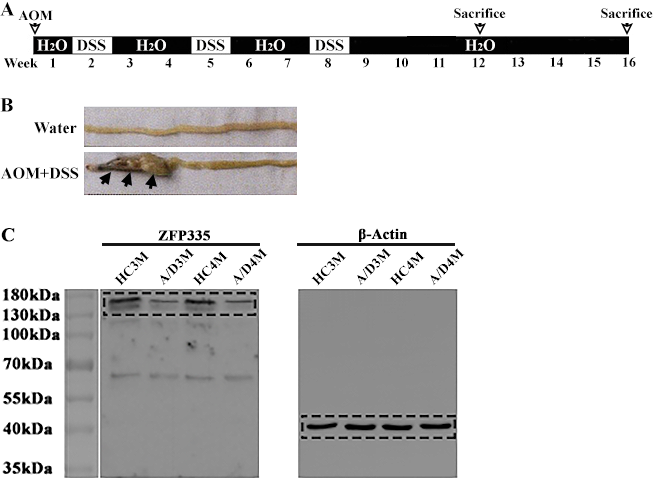


**Supplementary Fig. 1. CRC induction. (A)** Schematic of AOM plus DSS treatment. **(B)** Gross specimen photographs of tumor formation in the colon and rectum at month 3 (week 12). The arrows indicate tumors. **(C)** Uncropped images of Western blots displayed in Figure 1D.


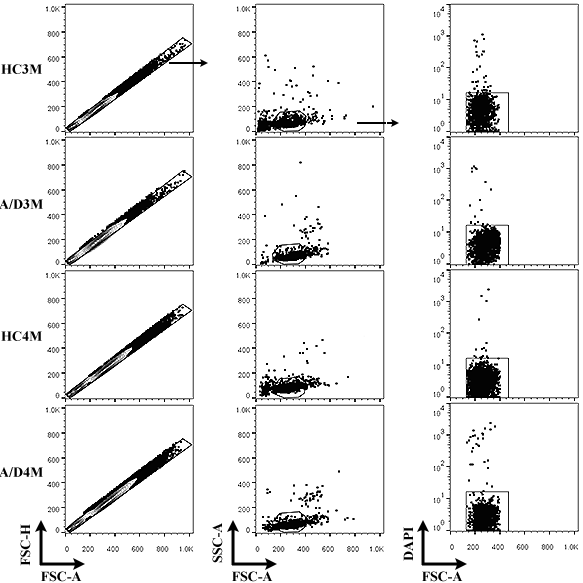


**Supplementary Fig. 2. Dot plots show the gating strategy for single cells, lymphocytes, and live lymphocytes isolated from mLNs.** HC3M: healthy mice at month 3. HC4M: healthy mice at month 4. A/D3M: 3 months after AOM/DSS treatment. A/D4M: 4 months after AOM/DSS treatment.


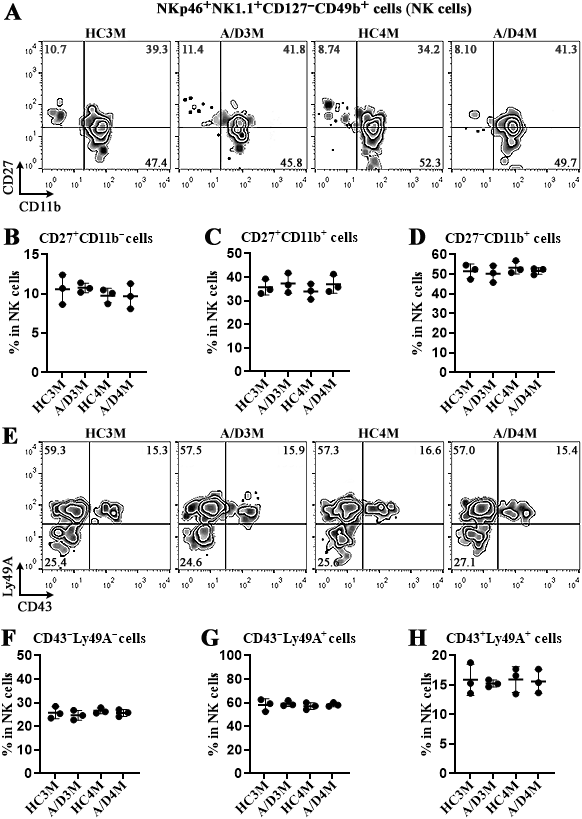


**Supplementary Fig. 3. The expression of CD27, CD11b, CD43, and Ly49A on mLN NK cells (NKp46^+^NK1.1^+^CD127^-^CD49b^+^ cells). (A)** Zebra plots show the expression of CD27 and CD11b. **(B to D)** Frequencies of indicated NK cell subsets. **(E)** Zebra plots show the expression of CD43 and Ly49A. **(F to H)** Frequencies of indicated NK cell subsets. HC3M: healthy mice at month 3. A/D3M: 3 months after AOM/DSS treatment. HC4M: healthy mice at month 4. A/D4M: 4 months after AOM/DSS treatment. N=3 mice per group in two experiments.


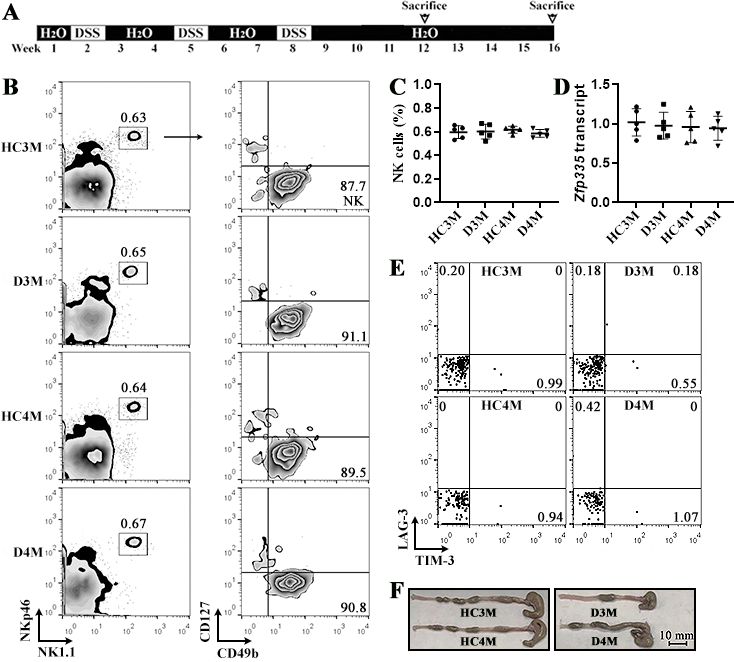


**Supplementary Fig. 4. ZFP335 expression in mLN NK cells after DSS treatment. (A)** Schematic diagram of DSS treatment. **(B)** Zebra plots show NKp46^+^NK1.1^+^CD127^-^CD49b^+^ NK cells in mLNs. HC3M: healthy mice at month 3. D3M: 3 months after DSS treatment. HC4M: healthy mice at month 4. D4M: 4 months after DSS treatment. **(C)** Frequency of mLN NK cells in total lymphocytes. **(D)** *Zfp335* transcript in mLN NK cells. N=5 mice per group in (C) and (D). **(E)** Dot plots indicate the expression of TIM-3 and LAG-3 on the surface of NKp46^+^NK1.1^+^CD127^-^CD49b^+^ NK cells. The plots represent two independent experiments. **(F)** Gross specimen photographs of colons.


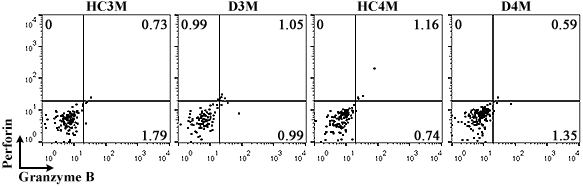


**Supplementary Fig. 5. Dot plots show intracellular perforin and granzyme B in mLN NKp46^+^NK1.1^+^CD127^-^CD49b^+^ NK cells after DSS treatment.** HC3M: healthy mice at month 3. D3M: 3 months after DSS treatment. HC4M: healthy mice at month 4. D4M: 4 months after DSS treatment. The plots represent three independent experiments.


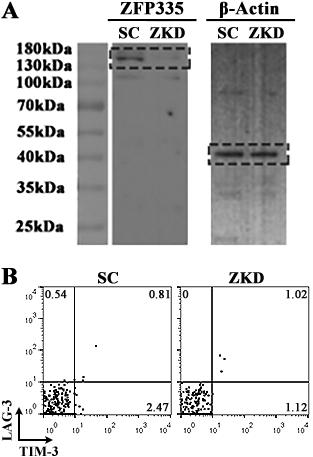


**Supplementary Fig. 6. (A) Uncropped images of Western blots displayed in Figure 4C.** **(B) Dot plots indicate the expression of TIM-3 and LAG-3 on the surface of splenic NK cells on day 3 after lentiviral transduction.** SC: transduction with scramble shRNA lentivirus. ZKD: transduction with ZFP335 shRNA lentivirus. The plots represent two independent experiments.


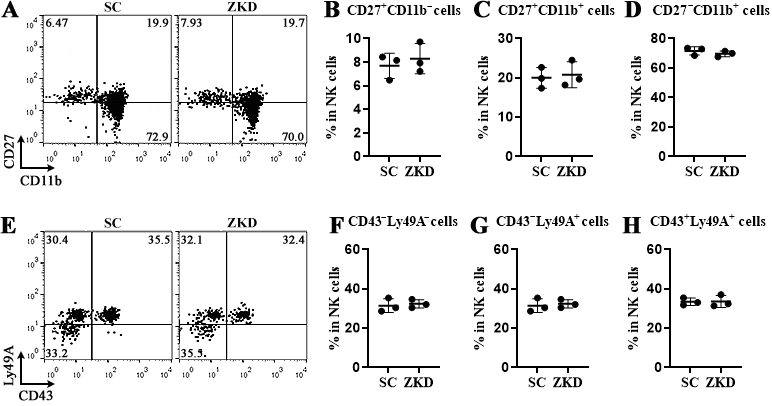


**Supplementary Fig. 7. The expression of CD27, CD11b, Ly49A, and CD43 on splenic NK cells on day 3 after lentiviral transduction. (A)** Dot plots show the expression of CD27 and CD11b. **(B to D)** Frequencies of indicated NK cell subsets. **(E)** Dot plots show the expression of Ly49A and CD43. **(F to H)** Frequencies of indicated NK cell subsets. SC: transduction with scramble shRNA lentivirus. ZKD: transduction with ZFP335 shRNA lentivirus. N=3 samples per group in two experiments.


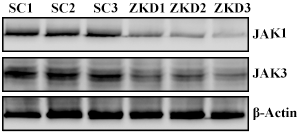


**Supplementary Fig. 8. Western blot images of JAK1 and JAK3 proteins in three independent batches of SC and ZKD NK cells, respectively.**
